# Cingulo-Opercular and Frontoparietal Network Control of Effort and Fatigue in Mild Traumatic Brain Injury

**DOI:** 10.3389/fnhum.2021.788091

**Published:** 2022-02-10

**Authors:** Amy E. Ramage, Kimberly L. Ray, Hannah M. Franz, David F. Tate, Jeffrey D. Lewis, Donald A. Robin

**Affiliations:** ^1^Interdisciplinary Program in Behavioral Neuroscience, Department of Communication Sciences and Disorders and Biological Sciences, University of New Hampshire, Durham, NH, United States; ^2^Department of Psychology, University of Texas, Austin, TX, United States; ^3^Department of Neurology, University of Utah School of Medicine, Salt Lake City, UT, United States; ^4^Mental Health Clinic, Wright Patterson Medical Center, Wright Patterson Air Force Base, Dayton, OH, United States

**Keywords:** brain injury – traumatic brain injury, fatigue, cognitive control networks, effort, neuroimaging

## Abstract

Neural substrates of fatigue in traumatic brain injury (TBI) are not well understood despite the considerable burden of fatigue on return to productivity. Fatigue is associated with diminishing performance under conditions of high cognitive demand, sense of effort, or need for motivation, all of which are associated with cognitive control brain network integrity. We hypothesize that the pathophysiology of TBI results in damage to diffuse cognitive control networks, disrupting coordination of moment-to-moment monitoring, prediction, and regulation of behavior. We investigate the cingulo-opercular (CO) and frontoparietal (FP) networks, which are engaged to sustain attention for task and maintain performance. A total of 61 individuals with mild TBI and 42 orthopedic control subjects participated in functional MRI during performance of a constant effort task requiring altering the amount of effort (25, 50, or 75% of maximum effort) utilized to manually squeeze a pneumostatic bulb across six 30-s trials. Network-based statistics assessed within-network organization and fluctuation with task manipulations by group. Results demonstrate small group differences in network organization, but considerable group differences in the evolution of task-related modulation of connectivity. The mild TBI group demonstrated elevated CO connectivity throughout the task with little variation in effort level or time on task (TOT), while CO connectivity diminished over time in controls. Several interregional CO connections were predictive of fatigue in the TBI group. In contrast, FP connectivity fluctuated with task manipulations and predicted fatigue in the controls, but connectivity fluctuations were delayed in the mild traumatic brain injury (mTBI) group and did not relate to fatigue. Thus, the mTBI group’s hyper-connectivity of the CO irrespective of task demands, along with hypo-connectivity and delayed peak connectivity of the FP, may allow for attainment of task goals, but also contributes to fatigue. Findings are discussed in relation to performance monitoring of prediction error that relies on internal cues from sensorimotor feedback during task performance. Delay or inability to detect and respond to prediction errors in TBI, particularly evident in bilateral insula-temporal CO connectivity, corresponds to day-to-day fatigue and fatigue during task performance.

## Introduction

Traumatic brain injury (TBI) was considered a *silent* epidemic in 2010 (Neurology, 2010) given a general lack of research and funding directed toward the understanding of its pathology and prognosis. This call to action from the field of neurology resulted in improved methods to detect injury (Mondello et al., 2014) and monitor recovery (Papadimitriou et al., 2016) from TBI, along with improved education and knowledge of the dangers of TBI, even in its mildest form. Nonetheless, rates of mild TBI are incredibly high, and because this mild form of injury is relatively invisible, i.e., it is not seen on neuroimaging metrics clinically and symptoms are unseen and require time to diagnose, it remains under-identified. Rates of mild TBI are particularly high in military service members given the nature of explosive munitions seen in deployments in support of Operation Enduring Freedom (OEF), Operation Iraqi Freedom (OIF), and Operation New Dawn (OND). The United States Defense Department has reported nearly 400,000 service members have been diagnosed with TBI since 2000, and nearly 9,000 in the first two quarters of 2021 (DoD Worldwide Numbers, 2021). Most of these injuries are mild (85.7%), but the fact that they are mild does not minimize the potential for long-term debilitating effects. Persistence of symptoms for longer than a month following mild TBI is termed post concussive syndrome, which may include fatigue, insomnia, pain, posttraumatic stress and others; all of which may have profound influences on quality of life (Losoi et al., 2015a). This syndrome has been documented at 6–12 month follow up sessions in the majority of a sample of 347 United States service members who suffered mild TBI while deployed (Mac Donald et al., 2017). Interestingly, the symptom of post concussive syndrome that is most strongly related to resilience is fatigue (Losoi et al., 2015b). While this symptom, in particular, has only been investigated in a few studies involving military service members (c.f., Ramage et al., 2019; Lewis et al., 2021), one study validates that persistent fatigue in this population is associated with poorer rates of return to productivity (Mortera et al., 2018). Thus, relationships among mild TBI, fatigue, and potential for recovery are intertwined and particularly prevalent in military service members.

Individuals with neurological disorders suffer from cognitive fatigue, which is known to impact cognitive performance. More specifically, cognitive fatigue is related to inability to sustain cognitive performance due to mental exhaustion, as opposed to inability to sustain physical exertion (Chaudhuri and Behan, 2000). However, neural dynamics of cognitive fatigue remain poorly understood. To gain an understanding of the neural substrates underlying cognitive fatigue, it is necessary to investigate the brain under conditions known to induce fatigue in health and brain injury, i.e., high cognitive demand, high sense of effort, and poor motivation. Each of these factors is governed, in part, by cognitive control systems that manage the ability to direct thought and action based on current goals and intentions. Intrinsic cognitive control networks are susceptible to injury given that they are diffuse and large-scale networks, and involve several brain regions known to be vulnerable in TBI (Bigler, 2017). Disruption of these networks is particularly apparent in individuals with mild traumatic brain injury (mTBI) in which cortical and subcortical axonal damage disrupts long-range connections that are essential to this level of higher cortical function (Han et al., 2016). It is well-known that structural network disruptions result in poor performance on tasks demanding cognitive control, and it is known that individuals with mTBI tend to get fatigued quickly in these types of tasks. In contrast, how cognitive control brain network function relates to fatigue is not explicitly known. The central hypothesis for this study is that mTBI impairs the coordination or integration of brain systems involved in the moment-to-moment monitoring, prediction, and regulation of behavior. This disruption in brain function results either in failure to motivate task engagement (Chaudhuri and Behan, 2000; Treadway et al., 2009; Botvinick and Braver, 2015; Sayalı and Badre, 2019), or mismatch between the sense of effort of an individual and the actual effort required to perform a task (Solomon et al., 1996; Coppieters et al., 2021; Lewis et al., 2021). In either case, the result is fatigue.

In a previous study on effort and fatigue in mTBI, we reported that functional connectivity between the left claustrum (anterior insula) and medial frontal and right-sided lateral inferior frontal regions is modulated by task-related changes in effort level. Furthermore, the strength of this connection is sensitive to fatigue to a greater extent in individuals with mTBI than control participants (Ramage et al., 2019). Specifically, immediate hyper-connectivity in the mTBI group was seen when task-related effort increased but connectivity strength diminished rapidly despite the need to maintain effort level over time. In contrast, similar patterns of connectivity were observed in the control participants, but only when there was a need to maintain higher levels of effort. These group differences were proposed as evidence of an inefficient brain system in the mTBI participants when task demand or fatigue is high.

In Ramage et al. (2019), the regions of interest for investigating functional connectivity were derived from task activation, which is one approach to seed-based correlational analyses in brain imaging data. However, given that known intrinsic connectivity networks (Fox and Raichle, 2007; Smith et al., 2009) are likely the scaffolding that supports healthy cognitive functioning, another approach is to investigate functional connectivity using collections of brain regions (or nodes) based on their associated intrinsic connectivity networks (Power et al., 2011). The latter method allows for testing hypotheses based on the established functional specialization of brain networks. As an example, with applicability to this study, the left claustrum is a node in the cingulo-opercular (CO) network, a cognitive control network associated with sustaining levels of (tonic) alertness (Sadaghiani and D’Esposito, 2015; Coste and Kleinschmidt, 2016). Sadaghiani and D’Esposito note that the CO network is sensitive to task demand, particularly when stimuli are unpredictable and require maintenance of alertness levels. Under these challenging conditions, the CO network is uniquely engaged relative to other cognitive control networks, i.e., the dorsal attention or frontoparietal (FP) network (Sadaghiani and D’Esposito, 2015). Further, CO connectivity is associated with generation of motor system force and visual-motor tracking tasks (Rinne et al., 2018) indicating a particular interaction between this brain network and the motor system. Unfortunately, that study did not remove the variance in CO connectivity associated with fatigue. As such, the task used in Ramage et al. (2019) was structured around motor performance, assessed decreasing performance associated with fatigue, and was used in the present study.

Additionally, Ramage et al. (2019) found that the strength of the left claustrum’s connections with the rostral and dorsal anterior cingulate cortex, medial frontal gyrus, and right inferior frontal gyrus (orbital and opercular) was correlated with fatigue evidenced by mTBI participants. While some of these additional functionally connected regions are considered part of the CO network, the others are more consistently considered to be within the FP network. The FP network governs cognitive control and flexibly modifies behavior based on task demands (Cole et al., 2013; Power and Petersen, 2013; Kao et al., 2020). In fact, Cole et al. (2013) demonstrated that the FP network has the highest global connectivity relative to other brain networks, and that it shifts its pattern of connectivity with other intrinsic brain networks depending on task demands. Investigations of the FP network relative to fatigue indicate that this network is particularly sensitive to time on task (TOT), and that increased TOT often results in failure of goal maintenance, error monitoring, and inhibition (Lim et al., 2016). The FP network is also central to learning, as it becomes more connected when engaged in novel contexts and decreases in connectivity as learning progresses (Buchel et al., 1999; Bassett et al., 2011; Sun et al., 2013; Kao et al., 2020).

Given the need to understand the neural bases of fatigue and their relationships to impaired performance in mTBI, we hypothesize that specific cognitive control networks are integrally involved in tasks that require not only tonic alertness or vigilance but also sustaining performance to meet changing task demands over time. As such, the CO and FP networks are also proposed to be primarily involved when there is a perception of high sense of effort and cognitive fatigue. In this study, we propose that (a) functional connectivity within each of these networks is weaker in mTBI, and (b) the functional connectivity of both networks is modulated by task demands (effort level and TOT) differently in the mTBI group relative to an orthopedic control group. We expect that the CO network may be more sensitive to TOT or fatigue through the duration of the task given its hypothesized role in generating the amount of force or tonic attention reported in previous studies. More specifically, we hypothesize that the CO network in the mTBI group will diminish in connectivity strength toward the end of each trial, as the need to maintain vigilance (TOT) and effort is required. Second, we expect that the FP network needs to be more strongly connected when there is a need to attain and maintain the level of effort to meet task demands, and that this coherence may be diminished in the mTBI group. We also predict that the extent of functional connectivity modulation associated with effort level or TOT will correlate with measures of state and trait fatigue. Finally, we expect that utilizing graph theoretical methods based on network neuroscience will better characterize brain dynamics underlying sense of effort and fatigue than basing connectivity metrics on task activation, as in Ramage et al. (2019).

## Materials and Methods

This manuscript presents a new analysis of data reported previously (Ramage et al., 2019) and is motivated by our previous findings that the left claustrum demonstrated modulated activity with varying effort levels, particularly in its connectivity with the medial and lateral frontal cortexes. Furthermore, this modulation of connectivity fluctuated in the mTBI participants with fatigue differently than that seen in the controls. These brain regions are component nodes in well-described cognitive control networks, i.e., the CO and FP networks, as described above. Thus, the analyses reported herein utilize the network-based statistic (NBS) (Zalesky et al., 2010) for the two cognitive control networks engaged for the task. Details of the task paradigm are described below.

### Participants

One hundred and three individuals who were referred to the TBI service at the San Antonio Military Medical Center in San Antonio, Texas (SAMMC) provided written informed consent for participation per an approved and monitored Institutional Review Board (No. 3743378) and Human Research Protection Office at the United States Army Medical Department Medical Research and Materiel Command (No. A-17660). Participants were excluded if using narcotic pain medications daily or had any MRI contraindications (i.e., metal in body, pregnancy). Sixty-one of these individuals made up the mTBI group, as defined by the VA/DoD Clinical Practice Guidelines for the Management of Concussion/mTBI. Injuries were sustained during deployment in support of OEF/OIF and occurred within 3–24 months of study participation. All the mTBI participants endorsed symptoms in at least three of the four cognitive clusters (somatic, sensory, affective, cognitive) on the Neurobehavioral Symptom Inventory (Cicerone and Kalmar, 1995). Injuries were sustained primarily from blast exposure (63%), other (20% that were blunt trauma, flash burns, and one gunshot wound), falls (10%) or motor vehicle accidents (7%). Forty-two control participants seen at SAMMC for orthopedic conditions served as controls in an effort to control for military-related traumatic experience (c.f., Wilde et al., 2019). More details about these participants may be found in Table 1 and in Ramage et al. (2019). Inclusion criteria for the control participants were: history of extracranial traumatic injury, age of 18–40, no history of mTBI in the past 3 years or any previous mTBI resulting in symptoms lasting longer than 48 h, no concurrent medical conditions (e.g., blindness) or neurological or psychiatric disorders, and no spinal cord injury resulting in loss of use in the upper extremities.

**TABLE 1:** Participant demographics.

	mTBI	Orthopedic Control
*n*	61	42
Age^#^	33 ± 10	37 ± 7
Sex (F:M)*	3:58	8:34
Years served^#^	11 ± 8	16 ± 6
Race		
White	46	29
Black or African American	7	12
American Indian or Alaskan Native	2	0
More than one race	6	1
Ethnicity		
Not Hispanic or Latino	42	34
Hispanic or Latino	18	8
Education*		
High school diploma/GED	36	10
Associate degree	13	12
College degree (BA/BS)	7	8
Post-graduate degree	4	12

*^#^Orthopedic Control > mild traumatic brain injury (mTBI); age: U(103) = 1655.5, p = 0.012, years served: U(103) = 1,783.4, p = 0.001.*

*More males than females in both groups (χ^2^_1_ = 5.2, p = 0.02).

*OC had higher education than mTBI (χ^2^_4_ = 17, p = 0.002).

### Mild Traumatic Brain Injury Comorbidities

The Symptom Checklist-90 Revised (Derogatis, 1994) is a 90-item self-report measure rated on a 5-point Likert scale. The participants rate their symptom experience, ranging from “not at all” to “extremely.” A raw score is converted to *t*-score utilizing normative data in the manual for men and women.

### Measure of Trait Fatigue

The Fatigue Severity Scale (FSS; Krupp et al., 1989) served as a proxy to trait fatigue, as it assesses self-rated levels of fatigue, and as it impacts motivation, activity level, and social participation. It is a nine-item questionnaire using a Likert scale (rating from 1-7) that documents the presence and severity of fatigue for the week prior to assessment. Scores range from 9 to 63, with higher scores indicating more severe effect of fatigue on daily life.

### Constant Effort and Measurement of State Fatigue

A constant effort (CE) task assessed sense of effort and fatigue, reported in detail in Ramage et al. (2019), requiring the participants to squeeze a pneumostatic bulb to a prescribed effort level (25, 50, or 75% of maximal effort level established prior to testing) and hold that effort level constant for 30 s. Each effort level trial was followed by 30 s of rest, and then repeated. A filtering algorithm was used to model the exponential function of the pressure on the bulb in real-time at each effort level. A median sliding window was used to identify the best solution for all the subjects. Time constant (TC) served as an index of state fatigue, characterizing the rate of decay for the sustained pressure on the bulb.

### MRI Data Acquisition and fMRI Preprocessing

Whole-brain MRI was acquired on a 3 Tesla Siemens Verio Sygno scanner (Siemens Medical Solutions, Malvern, PA, United States) at SAMMC. T1-weighted volumetric images were acquired for inspection of anatomy and spatial normalization (slice thickness = 1/0.5, TE/TR = 2.6/2,530, FOV = 256 mm, voxel size = 1 mm × 1 × 1 mm, 512 × 512 matrix, flip angle = 7°, and SENSE factor 2). Forty axial blood-oxygen-level-dependent (BOLD) echo planar slices were acquired during the CE task performance (slice thickness = 3/0.3 interleaved, FOV = 240 mm, voxel size = 3.43 mm × 3.43 mm × 3 mm, TE/TR = 30/2,500, flip angle = 90°, fold-over direction = AP, fat shift direction = P, and SENSE factor 2) for a total of 230 images acquired over a 9.6-min continuous scan. EPI images were corrected for slice timing and head movement by affine registration in SPM12.^1^ The mean EPI image for each subject was spatially normalized to the MNI template by unified segmentation (Ashburner and Friston, 2005). A deformation field was applied to the individual EPI images, and a 5-mm full width half mass (FWHM) Gaussian kerned smoothed the output images. Final spatial smoothing was then applied (8 mm, FWHM).

### Task-Related Functional Connectivity

Time courses were extracted for each subject for the 264 Power atlas nodes (Power et al., 2011). Sections of time courses associated with each effort level were excised so that 10 TRs of each level × 2 were concatenated for 2 blocks of each condition resulting in 20 TRs of data for each effort level. To address TOT, each effort level block was split into two, with a first half and a second half, each with 5 × 2 TRs of the data. For example, each effort level trial was 10 TRs such that at the 25% effort level, TRs 2 through 10 and 42 through 50 corresponded to the 25% trials for each participant, and so on. The mean time course for all nodes was then correlated. The task time course model was specified by the boxcar model described in supplementary material in Ramage et al. (2019). Pair-wise correlation (Pearson’s *r*) of the mean time course was calculated for all nodes, resulting in a 264-by-264 functional connectivity matrix. However, only connectivity among nodes in the CO (14 nodes) and FP (25 nodes) networks was assessed.

### Graph Theoretic Analysis

Network-Based Statistic (Zalesky et al., 2010) was used to test the hypothesized relationships between effort and fatigue and the brain networks associated with cognitive control (CO and FP). NBS is a statistical approach used to identify connections in a graph that are associated with changing psychological contexts during task performance. The advantage of NBS is that it tests the null hypothesis in interconnected components of edges rather than individually at each connection, such that the graphs are a collection of nodes linked together by a set of suprathreshold edges. Thus, NBS offers more power than the use of false discovery rate to identify networks of edges. Here, NBS was used to assess functional network differences for (a) main effects of group, effort level, and time-on-task (b), interactions among main effects, and (c) within-group effects of effort, time-on-task, and effort level-by-time-on-task. Effects for differences in functional networks were assessed across a range of *t*-thresholds to ensure reliability of results. Statistical thresholds for the analyses were: *F* > 40 or *t* > 2 (10,000 permutations), as these thresholds provide the most robust findings across a uniform threshold, and *p* < 0.05. Effect sizes for *t*-tests were calculated with Cohen’s *d*, as the average of significant *t*-values divided by the square root of *n*^2^.

Additionally, node degree, or the number of edges a node is connected with, was determined for each effect by network (i.e., main effects and interactions). Node degree can be used to identify “hubs” in the network and determine whether hubs differ depending on task manipulation or by group (Power et al., 2013). Also, edge length, or the Euclidean distance between nodes, was calculated as the square root of the sum of the squared differences between node coordinates. Edge length was correlated with FC to identify shared variance and group differences based on distance between nodes.

### Data Analysis

All the dependent variables were assessed for outliers and normality of distribution (Shapiro–Wilk test of normality) in SPSS (IBM Corporation, 2015). Age, years served, FSS scores, and TC were not normally distributed, and there was considerable variance within groups on the fatigue measures (Table 2). Thus, generalized mixed models were used to analyze group differences in these measures as well as contributions of other measures (depression, anxiety, and symptoms of posttraumatic stress disorder) to the variance in FSS or TC. These models allow for the accommodation of different distributions of the dependent variables as well as inclusion of random effects, i.e., participant to capture individual variability, and residuals. Comparisons of model fit (specifically the Akaike corrected information criterion, AIC) elucidated whether inclusion of random effects (participant and residual variance) improved the models. Model fit was compared using the AIC, and significant fixed effects were those with *p*-values < 0.05. *Post hoc* pairwise contrasts were controlled for multiple comparisons with the Bonferroni method to clarify significant fixed effects.

**TABLE 2:** Symptom Scales.

	mTBI	Orthopedic Control
Fatigue Severity Scale	39 ± 12	24.2 ± 9
PTSD Checklist – Military	49 ± 16	27 ± 14
**Symptom Checklist-90-R**		
Global Severity Index	1.3 ± 0.7	0.4 ± 0.6
Somatization	1.3 ± 0.7	0.6 ± 0.7
Obsessive compulsive	2.1 ± 0.9	0.7 ± 0.8
Interpersonal sensitivity	1.2 ± 0.8	0.3 ± 0.5
Depression	1.3 ± 0.8	0.4 ± 0.6
Anxiety	1.4 ± 0.9	0.3 ± 0.6
Hostility	1.4 ± 1	0.4 ± 0.6
Phobic anxiety	1.1 ± 0.8	0.2 ± 0.6
Paranoid ideation	1.1 ± 0.9	0.4 ± 0.6
Psychoticism	0.7 ± 0.7	0.2 ± 0.3

*Participants with mTBI reported more fatigue and symptoms of fatigue, obsessive compulsion, hostility, phobic anxiety, paranoid ideation, and psychoticism than the Orthopedic Controls. All U(102) > 300, p < 0.0001.*

Finally, functional connectivity for edges of each network with significant group, effort level, or time-on-task effects were added to the statistical models to identify their association with trait (FSS) and state (TC) fatigue for each group separately. Significant correlations were those with *p* < 0.05. In addition, group differences were assessed for each measure of fatigue with and without the inclusion of functional connectivity of the CO or FP edges. Edges were identified as potential predictors in the mixed models if they were significant in univariate general linear models (GLMs) (*p* < 0.05).

## Results

The controls were significantly older (*U* = 906, *n*1 = 61, *n*2 = 42, *p* = 0.012) and had higher levels of education (χ^2^_6_ = 19, *p* = 0.004) than the mTBI participants. There were more males than females in both groups (χ^2^_2_ = 6.9, *p* = 0.03). The groups were balanced for race (χ^2^_3_ = 7.4, *p* = 0.06) and ethnicity (χ^2^_1_ = 2, *p* = 0.21). The controls had more years of military service than the mTBI participants [*U*(1) = 738, *p* = 0.002, Table 1].

### Trait and State Measures of Fatigue

A generalized linear model was used to predict trait fatigue, measured by the FSS, and included group, age, depression, anxiety, and PCL-M scores as covariates or potential predictors. Model fit was best using gamma distribution with identity link. Group (β = 14.07, 95% CI 8.36–19.78), *p* < 0.001) and depression (β = 5.35, 95% CI 0.14–10.56), *p* < 0.044) were significant predictors indicating that the mTBI participants and those endorsing symptoms of depression had higher FSS scores.

**FIGURE 1:**
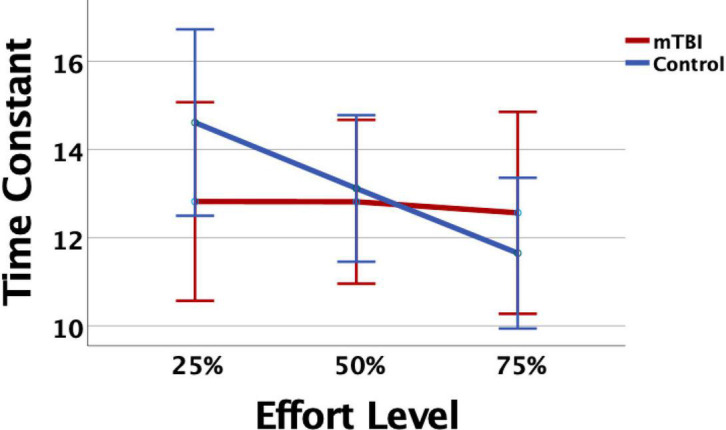
The mild traumatic brain injury (mTBI) and Orthopedic Control groups did not differ significantly in time constant (TC), but the Control group showed consistent decline, or evidenced fatigue, as effort level increased. Error bars represent 95% confidence intervals.

Measurement of task performance in the MRI scanner was susceptible to magnet-related noise, which affected the quality of CE data for some of the participants (Table 3). For the available data, variation in state fatigue indexed by TC was assessed using the generalized mixed model with repeated measures for effort level, and fixed effects for group, age, depression, anxiety, and PCL-M scores as covariates. A significant effort level effect indicated less fatigue (longer TC) in the 25% effort level (*b* = 0.24, 95% CI 0.04–0.44, *p* = 0.019). There was minimal change in TC across effort levels in the mTBI group, and the OC group demonstrated more fatigue at 50 and 75% effort levels (Figure 1), although the group-by-effort interaction was not significant. In addition, there were no significant effects of age, or presence of symptoms of depression, anxiety, or posttraumatic stress disorder. The random effect of participant was significant (*b* = 0.046, 95% CI 0.018–0.12, *p* = 0.04). Residual effects for effort level and TOT were also significant (all *p*s < 0.006), indicating that a large amount of variance in TC was not explained by the variables in the model.

**TABLE 3:** Constant effort task performance.

Effort level	Variable	mTBI	Orthopedic control
	*n*	21	23
25%	Actual pressure	29.7 ± 3.1	30.1 ± 3.7
	TC	13 ± 6	14 ± 7
	RMSE	0.79 ± 0.7	0.56 ± 0.24
	*n*	29	33
50%	Actual pressure	45.1 ± 5.9	51.2 ± 4.2
	TC	13 ± 6	12.9 ± 6
	RMSE	1.8 ± 1	1.4 ± 1
	*n*	18	31
75%	Actual pressure	55.3 ± 10.6	64.3 ± 5.4
	TC	13 ± 6	13 ± 6
	RMSE	2.9 ± 1.4	2.6 ± 1

*There were no significant group differences in average performance across the entire task or for each effort level. All the subjects increased pressure on the bulb (measured within the first 10 s of each trial) as instructed per effort level. Both groups tended to undershoot the prescribed effort level, although the mTBI group had a larger undershoot and more variance at the higher effort levels. RMSE, root mean square error of the time constant.*

### Cingulo-Opercular Network

#### Connectivity strength

**FIGURE 2:**
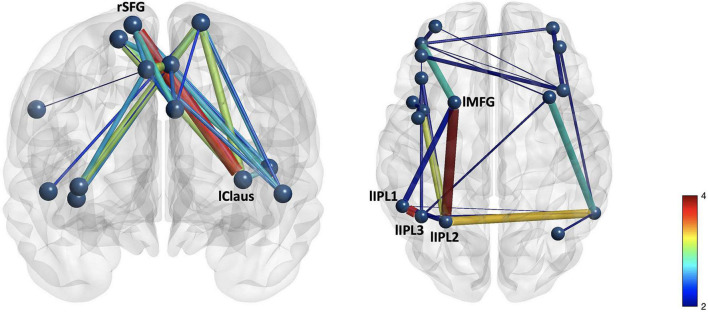
Group differences in **(A)** cingulo-opercular and **(B)** frontoparietal networks demonstrate stronger connectivity in the control group than in the mTBI group. The edge with the largest group difference in the CO network was the right superior frontal gyrus–left claustrum [*t*(103) = 3.76, *p* < 0.001, red]. Edges with the largest group differences in the FP network were the left inferior parietal lobule 1–left inferior parietal lobule 3 [*t*(103) = 3.73, *p* < 0.001], and left middle frontal gyrus 1–left inferior parietal lobule 2 [*t*(103) = 3.96, *p* < 0.001].

**FIGURE 3:**
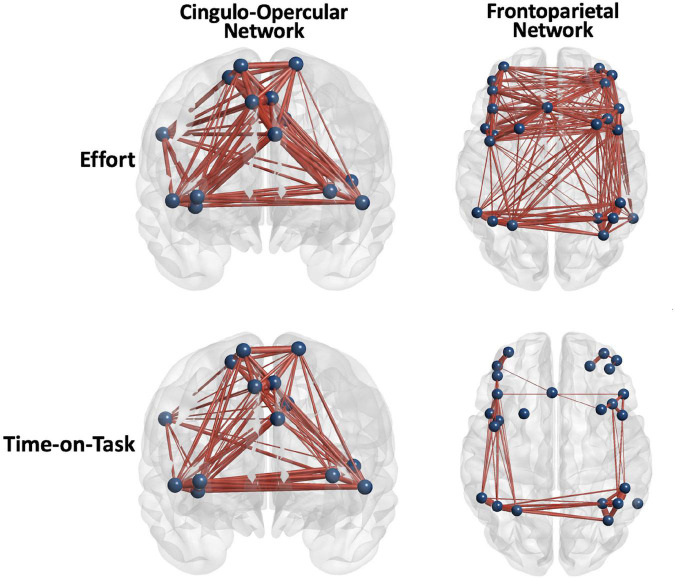
Positive main effects of effort level and time on task (TOT) for all the participants combined were robust, but the FP network was more diffusely engaged in effort level task manipulation. Edge thickness = *t*-value for the edge in the effect.

**FIGURE 4:**
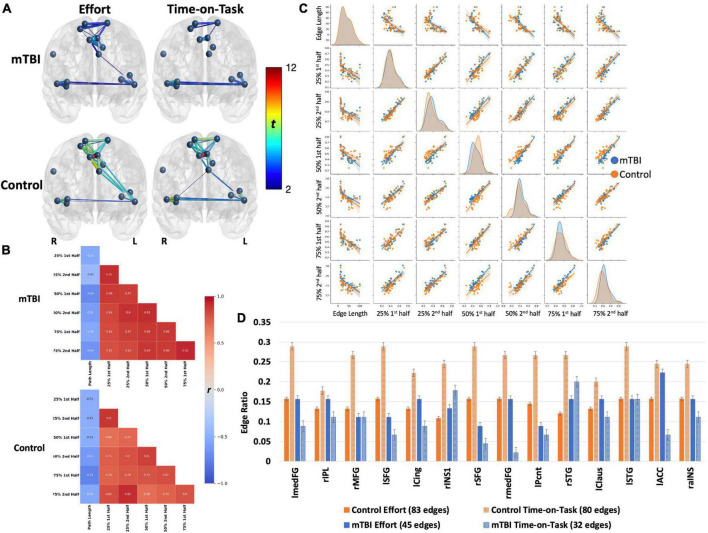
Both groups demonstrated robust main effects for effort level and TOT with similar spatial distributions across effects (effort or TOT) and across groups **(A)**. However, the effects were stronger in the control group (*t*-values presented by the color spectrum and edge thickness). For both groups, the edge with strongest effects (for effort and TOT) was the left medial frontal gyrus–right medial frontal gyrus [mTBI effort effect: *t*(63) = 5.24, control effort effect: *t*(40) = 11.81; mTBI TOT effect: *t*(63) = 4.61, control TOT effect: *t*(40) = 11.4, all *p*s < 0.001]. Connectivity between the left insular and medial frontal nodes was stronger for the effort effect in both groups, but only one edge connecting these brain regions was significant in the mTBI group (left precentral gyrus-left claustrum). Connections between medial frontal and insular or temporal nodes were absent in TOT for the mTBI group. The **(B)** heatmaps and **(C)** pairplots present correlation strengths and distributions (**C**, center diagonal) for the edges that were significant for effort level and TOT effects by group. The heatmaps show that for the controls (**B**, bottom), connectivity was strongest at the beginning of the task (25% effort level), indicated by darker red only in the top left corner, while connectivity was strong throughout the task in the mTBI group (**B**, top). The pairplots demonstrate that the FC over the course of the task was similar by group but more kurtotic in the OC group early in the task (at 25% second and 50% first). FC was more kurtotic at the end of the task for the mTBI group. The heatmaps and pairplots also present the negative relationship between edge length and FC in both groups, which was strongest at the 50 and 75% effort levels in the mTBI group, but more so for the 75% effort level in the OC group. The latter may indicate a “preference” for shorter Euclidean distances when a task becomes more effortful. **(D)** Node degree was presented as the number of connections each node was engaged in, standardized for the total number of significant edges in each effect (edge ratio), with more nodes engaged for the effect of effort level in the controls, but more nodes engaged in the TOT effect for the mTBI group. Error bars, standard error. lmedFG, left medial frontal gyrus; rIPL, right inferior parietal lobule; rMFG, right middle frontal gyrus; lSFG, left superior frontal gyrus; lCing, left mid cingulate cortex; rINS1, right insula 1; rSFG, right superior frontal gyrus; rmedFG, right medial frontal gyrus; lPcnt, left precentral gyrus; rSTG, right superior temporal gyrus; lClaus, left claustrum; lSTG, left superior temporal gyrus; lACC, left anterior cingulate cortex; and raINS, right anterior insula.

NBS analyses indicate that CO connectivity was stronger in the controls than in the mTBI participants [*t*(102) > 2.5, *p* < 0.001, Figure 2], although the effect size was small (*d* = 0.25). Significant group-by-effort [*F*(1, 101) > 40, *p* < 0.001 in 34 edges involving all 14 CO nodes] and group-by-time-on-task [*F*(1, 101) > 40, *p* < 0.001, in 32 edges, 14 nodes] interactions confirm that the CO network is engaged in both aspects of task performance, but differs by group (Figure 3). Within-group analyses were conducted to explore the nature of the significant interactions. Frequency distributions of connectivity strength among the nodes of the CO network by group and condition (Figure 4C pair plot, center diagonal) demonstrate that over the course of the task, CO FC peaked for most of the controls at the 50% first half [effort: average *t*(41) = 6.1, *p* < 0.001, *d* = 0.6 with 83 significant edges; time-on-task: average *t*(41) = 6.1, *p* < 0.001, *d* = 0.6 with 80 significant edges], and then decreased. Furthermore, the heatmaps and scatterplots in Figure 4 show that the correlation between the FC of the first and second halves of each trial was strong but diminished slightly over the course of the task and increasing effort level (25% *r* = 0.9, 50% *r* = 0.85, and 75% *r* = 0.8).

In contrast, CO connectivity for the mTBI group peaked later at the 75% effort level [Figure 4, mTBI effort: average *t*(62) = 3.2, *p* < 0.001, *d* = 0.32 with 45 significant edges, TOT: average *t*(62) = 4, *p* < 0.001, *d* = 0.39 with 32 significant edges], and connectivity of the CO network in both halves of each trial strongly correlated throughout the task (25% *r* = 0.91, 50% *r* = 0.91, 75% *r* = 0.93), suggesting little change in connectivity throughout the course of the task. As is also evident in Figure 2, there were fewer significant CO edges and less robust effects in the mTBI group relative to controls.

Spatial location of the edges associated with effort and time-on-task also varied across groups. Moderately strong edges connecting the medial frontal cortex to the insular cortex were above the threshold for the effort effect in the control group, but only one such connection was above the threshold for the mTBI group (i.e., left MCC – lSTG edge for effort effect only, and was one of the weaker edges, *t* = 2.25). Only one of these edges was also significant in the time-on-task effect in the controls, while none was significant in the mTBI group (Figure 2).

#### Network Organization

Along with the differences in CO connectivity strength between groups was some variation in the network organization as indexed by node degree. Twelve of the 14 CO nodes were involved in at least 20% of the edges for the controls, with higher degree for the time-on-task effect than the effort effect (Figure 4D). This means that the connectivity of the CO network was distributed across more nodes when there was a need to maintain a constant level of force on the bulb for the second half of the trials in the control group. In contrast, for the mTBI group, only the left MCC node was involved in >20% of the edges, and this was for the effort effect. In fact, more nodes were involved in the effort than time-on-task effects for 12 of the 14 nodes in the mTBI group. Only the right insula and right STG nodes demonstrated the pattern of node degree (i.e., time-on-task > effort) for the mTBI participants.

Connectivity was stronger in edges with smaller Euclidean distance among the significant edges, and was modified with effort level and time-on-task for both groups (Figure 2). In the control group, longer paths weakened in connectivity strength as effort level increased, particularly for the first half of the 75% effort level trials (*r* = −0.74). In the mTBI group, edges with longer paths had weaker connectivity strength for the 50 and 75% effort levels with little change over time.

### Frontoparietal Network

#### Connectivity Strength

**FIGURE 5:**
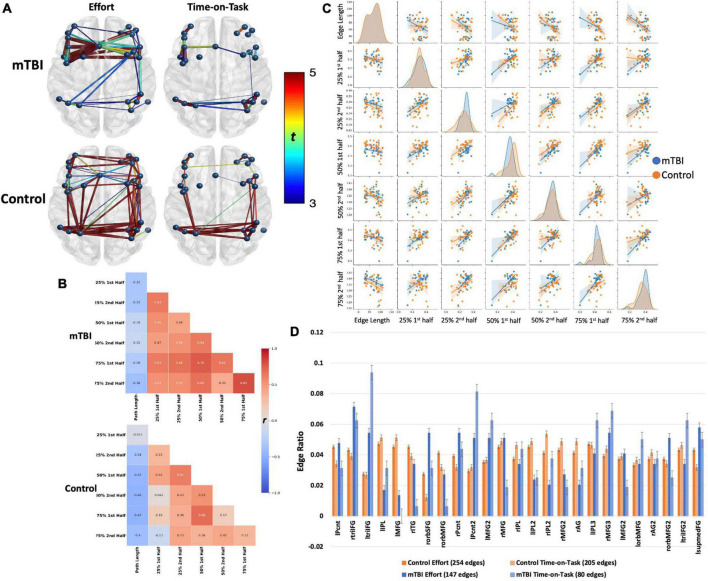
Frontoparietal network connectivity by group. Both groups demonstrated robust main effects for effort level and TOT with similar spatial distributions across effects (effort or TOT) and across groups. The effects were stronger in the control group (*t*-values presented by the color spectrum) than in the mTBI group but with small effect size (*d* = 0.25). Spatial distribution was larger for the effort level than for TOT in both groups, but effect sizes did not differ (mTBI *d* = 0.31 for both, control *d* = 0.41 for effort, *d* = 0.4 for TOT). However, on visual inspection of the network, the distribution of significant edges and stronger edges (*t*-values presented by color spectrum and edge thickness), was greater among the frontal nodes than the parietal nodes in the mTBI group. In contrast, connectivity was well distributed across nodes in the control group. This pattern, i.e., more and stronger connections in frontal than parietal nodes, is also evident in the TOT effect. The **(B)** heatmaps and **(C)** pairplot present the correlation strengths and distributions (**C**, center diagonal) for edges that were significant at the effort level and TOT effects by group, and the scatter plots present the relationships between each of the effort levels by TOT. The heatmaps demonstrate that the mTBI group (**B**, top) had stronger FC throughout the task with only slight increases in FC for the first half of each trial. In contrast, the connectivity of the controls varied consistently for the first half of the 50 and 75% effort levels. The pairplot presents the distributions of FP connectivity strength for the significant edges by group, and while the NBS results did not indicate strong group differences in the FP (evident in **A**), these plots make evident the differing patterns of FC relationships by group. First, the distributions differ in the OC by TOT, with a more kurtotic peak for the first trial at 25% that flattens in the second half at 25%, and then remain near the same for the other effort levels with slight skew to the right (stronger connectivity). This was not the case in the mTBI group where the distribution was kurtotic for all effort levels. In both groups, there were bimodal distributions, with smaller numbers of participant data skewed to the left (weaker connectivity). Also evident in the **(B)** heatmaps is the negative correlation of edge length with FC. This negative correlation was slightly weaker in the mTBI group, and there was a trend toward more negative FC path length correlations with increasing effort level in the OC group. **(D)** Node degree, presented as the number of nodes by the total number of edges for each effect and group, demonstrated group differences in the numbers of connections each node was engaged in, with higher degree in frontal nodes for the mTBI group relative to the control group. The involvement of frontal nodes was particularly higher for the mTBI group in the TOT effect. lPcnt, left precentral gyrus; rtriIFG, right inferior frontal gyrus triangularis 1; ltriIFG, left inferior frontal gyrus triangularis 1; lIPL, left inferior parietal lobule 1; lMFG, left middle frontal gyrus 1; rITG, right inferior temporal gyrus 1; rorbSFG, right superior frontal gyrus orbitalis 1; rorbMFG, right middle frontal gyrus orbitalis 1; rPcnt, right precentral gyrus 1; lPcnt2, left precentral gyrus 2; lMFG2, left middle frontal gyrus 2; rMFG, right middle frontal gyrus 1; rIPL, right inferior parietal lobule 1; lIPL2, left inferior parietal lobule 2; rIPL2, right inferior parietal lobule 2; rMFG2, right middle frontal gyrus 2; rAG, right angular gyrus 1; lIPL3, left inferior parietal lobule 3; rMFG3, right middle frontal gyrus 3; lMFG2, left middle frontal gyrus 2; lorbMFG, left middle frontal gyrus orbitalis 1; rAG2, right angular gyrus 2; rorbMFG2, right middle frontal gyrus orbitalis 2; ltriIFG2, left inferior frontal gyrus triangularis 2; lsupmedFG, left superior medial frontal gyrus.

Frontoparietal connectivity was significantly stronger in the controls than in the mTBI group, although the effect size was very small [*t*(102) = 2, *p* < 0.001, *d* = 0.14, Figure 2]. The FP network was robustly modulated by effort level [*t*(102) > 5, *p* < 0.001, *d* = 0.73] and TOT [*t*(102) > 5, *p* < 0.001, *d* = 0.76], primarily in intra- and inter-parietal edges as well as intra-frontal edges on the right (effort effect) and left (TOT effect) (Figures 3, 5). FC increased in the control group at the 25 and 50% effort levels but decreased at the 75% effort level [effort: average *t*(102) = 5.3, *p* < 0.001, *d* = 0.52 with 254 significant edges]. However, at each effort level, FC was greater in the first than in the second half of trials [time-on-task: average *t*(102) = 6, *p* < 0.001, *d* = 0.6 with 205 significant edges].

Conversely, connectivity increased considerably at the 50% effort level in the mTBI group, only minimally changed with time-on-task, and remained strong throughout the 75% effort trials [effort: average *t*(102) = 5.3, *p* < 0.001, *d* = 0.52 with 147 significant edges; time-on-task: average *t*(102) = 3.8, *p* < 0.001, *d* = 0.37 with 80 significant edges]. Correlations between the first and second half of the trials further support that the FP network was modulated to a larger degree for time-on-task in the controls (i.e., weak correlations between halves in the controls, moderate to strong correlations in the mTBI group).

#### Network Organization

Frontoparietal node degree indicated distributed connectivity across all the 25 nodes, with no single node present in more than 10% of the edges for either group. However, unlike the CO network, there were frontal nodes with higher node degree in the mTBI group than in the control group, particularly for the time-on-task effect, i.e., the left inferior frontal triangularis, and precentral, middle frontal, and right middle frontal nodes. As with the CO network, the mTBI group had fewer significant edges between frontal and parietal nodes for both the effort level and time-on-task effects (Figure 5A).

Also evident was stronger connectivity with shorter path lengths. This was particularly evident with increasing effort level in the control group. However, the strength of the relationship between path length and frontoparietal FC was minimal (all *r* < −0.5 in the controls, all *r* < −0.4 in the mTBI group) (Figure 5B).

### Predicting Trait and State Fatigue With Cingulo-Opercular or Frontoparietal Functional Connectivity Strength

Edges with significant group-by-effort and/or group-by-TOT effects for all the networks using NBS were then assessed for their relationship with measures of trait (FSS) or state (TC) fatigue using univariate GLMs. Edges found to be significant predictors of one of the fatigue measures in the univariate GLMs were then added to the generalized linear mixed models, along with group, experimental condition (effort level or TOT), or demographic variables (e.g., age, depression, etc.) that were also predictive of FSS or TC. Using a stepwise method, non-significant variables were removed (*p* < 0.05) to determine the model of best fit.

#### Fatigue Severity Scale: Trait Fatigue

The final generalized linear model to predict FSS for the entire sample (98 participants who had FSS scores) included group (β = 0.536, 95% CI 0.39–0.67, *p* < 0.001) and FP edge strength for the rITG-rMFG2 edge at the 75% effort level, second half (β = −0.33, 95% CI −0.53 to −0.12, *p* < 0.002), indicating that those in the mTBI group and those with weaker FC of that edge during the second half of the 75% effort level trials had higher FSS scores (likelihood ratio χ^2^_2_ = 46.52, *p* < 0.001, gamma with log link).

Prediction of FSS including edges in the model yielded very different results in each group considered separately. In the mTBI group, the model with best fit (Table 4) included depression (β = 0.079, 95% CI 0.012–0.146, *p* < 0.02), five edges of the CO network: stronger connectivity of lACC-raINS (β = 0.239, 95% CI −0.042 to 0.435, *p* = 0.017), lmedFG-rSFG (β = 0.316, 95% CI 0.085–0.547, *p* = 0.007), rIPL-lSFG (β = 0.191, 95% CI 0.024–0.357, *p* = 0.025) for the second half of the 50% effort trials, weaker connectivity of rINS1-lSTG (β = −0.244, 95% CI −0.415 to −0.073, *p* = 0.005), and rSTG-lSTG (β = −0.332, 95% CI −0.511 to −0.153, *p* < 0.0001), and FP edge strength for rMFG3-lorbMFG (weaker, β = −0.274, 95% CI −0.47 to −0.077, *p* = 0.006) and rMFG3-lMFG2 (stronger, β = 0.398, 95% CI 0.203–0.593, *p* < 0.0001). This finding indicates involvement of both the CO and FP networks, and more specifically weaker FC involving the left STG node of the CO network, in trait fatigue for the mTBI group.

**TABLE 4:** Significant predictors of Fatigue Severity Scale (FSS) in each group based on the generalized linear model with gamma (log) distribution for the FSS.

Network	Parameter	*B*	95% CI		Hypothesis test		
			Lower	Upper	Wald Chi-square	df	*P*
**mTBI group**							

	(Intercept)	3.404	3.206	3.602	1137.31	1	<0.001
	Depression	0.079	0.012	0.146	5.38	1	0.02
Cingulo-opercular	50% second rINS1-lSTG	−0.244	−0.415	−0.073	7.844	1	0.005
	75% first rSTG-lSTG	−0.332	−0.511	−0.153	13.146	1	<0.001
	50% second rIPL-lSFG	0.191	0.024	0.357	5.017	1	0.025
	50% second lACC-raINS	0.239	0.042	0.435	5.66	1	0.017
	50% second lmedFG-rSFG	0.316	0.085	0.547	7.189	1	0.007
Frontoparietal	75% second rMFG3-lorbMFG	−0.274	−0.47	−0.077	7.412	1	0.006
	75% second rMFG3-lMFG2	0.398	0.203	0.593	16.003	1	<0.001
	(Scale)	0.048	0.034	0.069			

**Orthopedic controls**							

	(Intercept)	2.42	1.833	3.008	65.225	1	<0.001
Cingulo-opercular	50% first lPcnt-lSTG	0.557	0.279	0.835	15.387	1	<0.001
	50% second rSTG-raINS	0.567	0.155	0.979	7.27	1	0.007
Frontoparietal	75% first lIPL3-rAG2	−0.444	−0.798	−0.091	6.069	1	0.014
	75% first rAG-rAG2	−0.385	−0.727	−0.043	4.876	1	0.027
	75% first lPcnt2-lIPL2	−0.366	−0.66	−0.072	5.951	1	0.015
	75% first lPcnt-ltriFG2	−0.268	−0.54	0.005	3.712	1	0.054
	75% first lIPL-rMFG2	−0.026	−0.554	0.035	2.99	1	0.084
	75% first lIPL2-ltriFG2	0.364	0.04	0.689	4.839	1	0028
	75% first lMFG2-rMFG3	0.376	0.168	0.584	12.524	1	<0.001
	75% first lIPL2-rAG2	0.502	0.196	0.807	10.377	1	0.001
	75% first lIPL-lMFG	0.572	0.228	0.916	10.629	1	0.001
	75% first rIPL-rIPL2	0.739	0.262	1.216	9.223	1	0.002
	75% second rIPL-rAG2	−0.782	−1.095	−0.468	23.896	1	<0.001
	(Scale)	0.049	0.032	0.0076			

*l, left; r, right hemisphere; 2 or 3 indicate multiple nodes within the same gyrus (see Supplementary Table 1 for coordinates and labels for the nodes); ACC, anterior cingulate cortex; AG, angular gyrus; aINS, anterior insula; INS, insula; IPL, inferior parietal lobule; medFG, medial frontal gyrus; MFG, middle frontal gyrus; orbMFG, orbital middle frontal gyrus; Pcnt, precentral gyrus; SFG, superior frontal gyrus; STG, superior temporal gyrus; triFG, triangularis frontal gyrus.*

The connectivity of both networks, particularly the FP network, at the 75% effort level, was associated with FSS in the Control group. The stronger connectivity strength of two CO edges at the 50% effort level (lPcnt-lSTG: β = 0.557, 95% CI 0.279–0.825, *p* < 0.0001 and rSTG-raINS β = 0.567, 95% CI 0.155–0.979, *p* = 0.007) and weaker connectivity for six edges and stronger connectivity for five edges of the FP were significant predictors (Table 4). Depression was not a significant predictor of FSS in the Control group.

#### Time Constant During the Constant Effort Task – State Fatigue

Univariate GLMs were also used to identify CO and FP edges that were potential predictors of state fatigue (TC). These were added to the generalized mixed model with repeated effect of effort level and fixed effect of group. Non-significant predictors were removed. The final model (Table 5) indicated a significant interaction of group-by-edge for the lIPL–rIPL2 and rIPL2–rIPL3 edges of the FP network, as well as significant fixed effects for additional edges (rAG–lIPL3 and rMFG2–lIPL3) and effort level (25% effort level was less fatiguing than 50 or 75%). Also, the random effect of participant indicated considerable individual variability. As with the initial model without network edges, the residual effects were significant, indicating that a large amount of variance in TC was not explained by the model.

**TABLE 5:** Significant predictors of time constant (TC) in each group based on the generalized mixed model with gamma (log) distribution for TC, compound variances assumed for the random covariance, and diagonal covariance structure assumed for the residual covariance.

	mTBI					Control				
		*b*	95% CI		*p*		*b*	95% CI		*p*
Fixed effects										
Intercept		2.6	2.5	2.7	0.00		2.3	2.1	2.6	0.00
Effort level										
25%							25%	0.6	0.3	0.9
50%							50%	0.2	0.0	0.4
Time on task							0.2	0.0	0.4	0.05
Edge										
	CO_rSTG-lClaus	−0.2	−0.5	0.0	0.05	CO_lSFG-rmedFG	0.2	0.0	0.4	0.10
						FP_llIPL-rIPL2	0.6	0.3	1.0	0.00
						FP_rAG-lIPL3	0.4	0.1	0.7	0.01
						FP_rMFG2-lIPL3	−0.2	−0.5	0.0	0.03
						FP_lIPL2-lIPL3	−1.1	−1.4	−0.7	0.00
Effort level × time on task										
						[Effort = 25] × [Half = 1]	−0.6	−0.9	−0.2	0.00
						[Effort = 50] × [Half = 1]	−0.2	−0.5	0.1	0.26
Random effect covariance										
Participant		0.0	0.0	0.2	0.24		0.1	0.0	0.1	0.0
Residual effects										
	[Effort = 25] × [Half = 1]	0.2	0.1	0.5	0.04	[Effort = 25] × [Half = 1]	0.1	0.0	0.3	0.06
	[Effort = 25] × [Half = 2]	0.1	0.0	0.2	0.02	[Effort = 25] × [Half = 2]	0.2	0.1	0.4	0.01
	[Effort = 50] × [Half = 1]	0.1	0.1	0.3	0.01	[Effort = 50] × [Half = 1]	0.2	0.1	0.3	0.00
	[Effort = 50] × [Half = 2]	0.2	0.1	0.5	0.01	[Effort = 50] × [Half = 2]	0.1	0.1	0.2	0.00
	[Effort = 75] × [Half = 1]	0.3	0.1	0.8	0.02	[Effort = 75] × [Half = 1]	0.2	0.1	0.3	0.00
	[Effort = 75] × [Half = 2]	0.2	0.1	0.5	0.01	[Effort = 75] × [Half = 2]	0.1	0.1	0.3	0.01

*l, left; r, right hemisphere; 2 or 3 indicate multiple nodes within the same gyrus (see Supplementary Table 1 for coordinates and labels for nodes); ACC, anterior cingulate cortex; AG, angular gyrus; Claus, claustrum; IPL, inferior parietal lobule; medFG, medial frontal gyrus; MFG, middle frontal gyrus; STG, superior temporal gyrus.*

Again, the groups considered separately elucidated differing relationships between connectivity and TC. In the mTBI group, recall that TOT did not vary for TC in the participants for whom the data were available (*n* = 100 observations across 31 participants). The only significant predictor of TC was weaker connectivity of CO edge rSTG-lClaus (β = −0.226, 95% CI −0.451 to −0.001, *p* = 0.049). Effort level and time-on-task were not significant predictors. Residual effects were significant for all effort by TOT modulations, but the random effect of participant was not significant.

**FIGURE 6:**
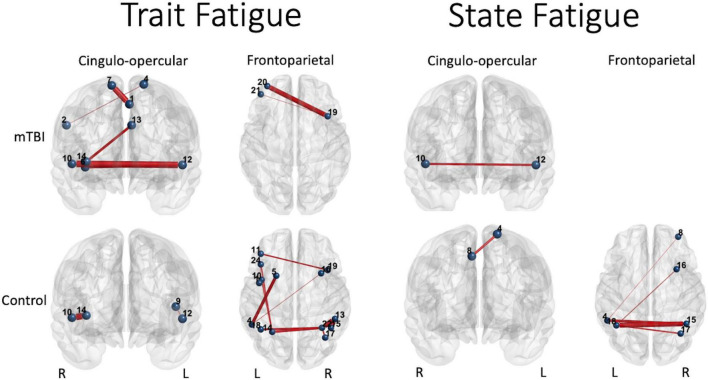
Significant predictors (edge thickness = *b* coefficient size) of trait (FSS) or state (TC) fatigue tended to involve edges of the CO network in the mTBI group (top row), but not the FP network in the control group (bottom row). The connectivity of several CO edges was predictive of trait fatigue (FSS score) in the mTBI group, specifically weaker connectivity of two edges in the latter part of the 50% effort level second half or 75% effort level first half, and stronger the connectivity of three edges during the most effortful (50 and 75% effort levels) in the second half of the trials (longer TOT). In addition, two FP edges in the highest effort level (75%) at longer TOT (second half) were predictive of trait fatigue in the mTBI group. In contrast, it was largely FP edge connectivity, and specifically the parietal edges, along with only two edges of the CO, that were predictive of trait fatigue in the OC group. Similar group differences were seen for prediction of state fatigue (time constant during the constant effort task), with several FP edges predicting fatigue in the OC group. Interestingly, the weaker connectivity between the right superior temporal gyrus (Mortera et al., 2018) and the left claustrum (Bigler, 2017) in the mTBI group predicted more trait and state fatigue. Note: Edge thickness indicates predictive strength (absolute beta value) from the generalized linear model predicting FSS score (reported in Table 4) or the generalized mixed model predicting TC (reported in Table 5). Cingulo-opercular network: 1, left medial frontal gyrus; 2, right inferior parietal lobule; 3, right middle frontal gyrus; 4, left superior frontal gyrus; 5, left mid cingulate cortex; 6, right insula 1; 7, right superior frontal gyrus; 8, right medial frontal gyrus; 9, left precentral gyrus; 10, right superior temporal gyrus; 11, left claustrum; 12, left superior temporal gyrus; 13, left anterior cingulate cortex; and 14, right anterior insula. Frontoparietal network: 1, left precentral gyrus; 2, right inferior frontal gyrus triangularis 1; 3, left inferior frontal gyrus triangularis 1; 4, left inferior parietal lobule 1; 5, left middle frontal gyrus 1; 6, right inferior temporal gyrus 1; 7, right superior frontal gyrus orbitalis 1; 8, right middle frontal gyrus orbitalis 1; 9, right precentral gyrus 1; 10, left precentral gyrus 2; 11, left middle frontal gyrus 2; 12, right middle frontal gyrus 1; 13, right inferior parietal lobule 1; 14, left inferior parietal lobule 2; 15, right inferior parietal lobule 2; 16, right middle frontal gyrus 2; 17, right angular gyrus 1; 18, left inferior parietal lobule 3; 19, right middle frontal gyrus 3; 20, left middle frontal gyrus 3; 21, left middle frontal gyrus orbitalis 1; 22, right angular gyrus 2; 23, right middle frontal gyrus orbitalis 2; 24, left inferior frontal gyrus triangularis 2; and 25, left superior medial frontal gyrus.

In contrast, effort level by time-on-task interacted for the Control group, with TC being longest in the first half of the 25% effort level and diminishing (1) with increasing effort level and (2) in the second half of each trial, Also, the connectivity of one CO edge (stronger rmedFG-lSFG connectivity) and four FP edges (two stronger, two weaker) involving the bilateral parietal nodes and one frontal node was predictive of TC (Table 5 and Figure 6).

## Discussion

The results from this study inform the neural organization of effort and fatigue in individuals with mTBI relative to controls. The functional connectivity of the CO and FP networks during the CE task performance was generally weaker in the mTBI group than in the control group. However, the dynamics of connectivity change over the course of the task elucidated slight variations between groups, particularly for maintaining connectivity with longer TOT. Increasing effort level corresponded to increased connectivity strength for both networks in both groups, but the mTBI group demonstrated stronger FC later in the task (particularly for the CO) and sustained stronger connectivity for the entirety of the task (both networks) than the controls. More striking was that FP connectivity fluctuated with TOT (first half > second half), i.e., modulated to meet task demands, in the control group while mTBI FP connectivity did not. Importantly, the timing of those fluctuations, and network associated with them by group appear most predictive of self-report trait fatigue, as well as fatigue measured during task performance.

### Network Connectivity and Organization

The groups did not differ for state fatigue during task performance, similar to data reported by Prak et al. (2021) who used a similar task in an fMRI study in mTBI versus healthy controls. Also consistent with the data reported by Prak et al. (2021) were only minimal differences in brain network connectivity (Figure 2) with small effect sizes. The largest differences in CO connectivity were between medial frontal and anterior insula or claustrum nodes, and FP group differences were primarily in edges connecting the parietal nodes to middle frontal or inter-parietal nodes. In both networks, there was a reduction in the number of edges connecting the frontal nodes to the temporal nodes (for the CO) or parietal nodes (for the FP) in the mTBI group. These findings are consistent with those reported by others in cognitive control networks (e.g., Han et al., 2016; Philippi et al., 2021) as well as in other intrinsic brain networks (e.g., default mode network, Santhanam et al., 2019; Philippi et al., 2021). These regional disconnections, separating frontal from either temporal or parietal nodes, provide additional evidence for the disconnection hypothesis of Fagerholm et al. (2015) that network “hubs” (nodes within a network that efficiently connect other nodes) are disconnected in TBI. Importantly, the degree of disconnection (reduced betweenness centrality) of the hubs is predictive of impaired cognitive performance (Fagerholm et al., 2015). While hubs were not identified in the CO and FP networks as part of the analyses reported here, the edge ratios for each network (number of connections each node is engaged in divided by the total number of edges, Figures 4D, 5D) indicated greater involvement of frontal nodes in the mTBI group relative to the controls for both networks, suggesting that frontal nodes may represent hubs in the mTBI group that are disconnected from their neighboring nodes with longer path lengths. The latter hypothesis would be best assessed with other graph theory metrics (e.g., betweenness, modularity) in future studies.

### Task Modulation of Network Connectivity

Unlike network organization, group differences were most apparent in each network for the presence, or not, of task-related modulation of network connectivity strength. The TC for the CE task indicated that the mTBI participants maintained constant pressure on the bulb throughout the trials without decay over time, and did not vary by effort level (Figure 1), unlike the controls. This was also essentially the pattern of findings for the CO network, that network connectivity demonstrated task-related modulation in the control group for effort level and TOT but was relatively constant and strong throughout the task in the mTBI group. The FP connectivity pattern fluctuated even more with effort level in the control group, while it remained particularly strong only for the higher effort level trials (50 and 75%) in the mTBI group without clear TOT modulation. The group differences in the timing of peak connectivity, particularly of the FP, corresponding to peak effort level or longer TOT, suggest that the mTBI group has hyper-connectivity of the CO, or hypo-connectivity of the FP network, either of which is different from that seen in the control group.

That the temporal dynamics of brain network activity differs in the individuals with TBI is a fairly recent discovery, reported in resting state fMRI (Vergara et al., 2017, 2018; Hou et al., 2019; Lu et al., 2021), in task-related fMRI (Gilbert et al., 2018), and in magnetoencephalographic resting state recordings (Antonakakis et al., 2019). In all of those examples, the differences in mTBI tended to be in frontal or parietal nodes and are likely specific to certain frequency band oscillations (beta frequency in Antonakakis et al., 2019). Similarly, network “states,” i.e., finite sets of coactivation patterns carrying different connectivity characteristics, have been described to demonstrate changes in network organization over time that are aberrant in TBI, with fewer transitions between states relative to healthy controls (Gilbert et al., 2018; Vergara et al., 2018). Importantly, the temporal configuration of brain networks changes with the evolution of recovery in mTBI (from acute to sub-acute, Vergara et al., 2017; Hou et al., 2019) and is associated with persistent, chronic cognitive symptoms (Prak et al., 2021). These studies have been conducted by whole brain analyses and identified important differences in brain dynamic network states. For example, state 2 in the Vergara et al. study (Vergara et al., 2018) had significant but sparse changes in connectivity strength in the mTBI group relative to the control group, involving the paracentral cortex/supplementary motor area and cerebellar nodes. Those changes discriminated the groups with 92% accuracy. The findings reported here centered only on the CO and FP networks, and thus cannot be directly compared to the other studies of whole brain network dynamics, but nonetheless suggest that while overall network connectivity differences are minimal between mTBI and controls, the shifting of network organization with task modulation is considerably different and may be clinically relevant. For example, the hyper-connectivity of the CO network and hypo-connectivity of the FP network in the mTBI group may reflect an adaptive or compensatory brain response to increased need to sustain effort. This adaptation to the brain response is successful in that TC did not demonstrate decay/fatigue in the mTBI group relative to the controls but may come at a cost: fatigue. Such compensatory or adaptive neural responses have been documented in other populations that are vulnerable to fatigue (c.f., Dobryakova et al., 2015, 2016).

### Network Connectivity Relative to Trait and State Fatigue

Specific to the presence of symptoms of fatigue, the relationships between FC and state or trait fatigue measures suggest group-specific roles of both networks relative to fatigue. The mTBI participants reported significantly more trait fatigue on the FSS than the controls. The FSS scores were predicted, in part, by the presence of depression in the mTBI group, but also primarily by CO edge strength at the 50 and 75% effort levels when there was a need to maintain effort over time (longer TOT). In contrast, it was FP edge strength at the 75% effort level, irrespective of TOT, that predicted FSS scores in the controls. This finding suggests that the over-connectivity of the CO network utilized to attain and maintain effort during task performance is associated with the self-perceived feeling of fatigue in these participants. For the control group, it was FP connectivity that was needed to attain the goal (75% effort) that associated with FSS score. While it is presumptive to assume that task performance on 1 day while in the scanner is representative of day-to-day activity relating to the construct of fatigue that the FSS represents, these findings suggest that the adaptation of brain organization to rely on the CO network for monitoring and flexibly navigating the need for more effort is inefficient and, in turn, relates to elevated perceived trait fatigue.

State fatigue, as indexed by the decay in pressure over time (TC) while performing the CE task, was associated only with CO connectivity, or more precisely connectivity of one CO edge, in the mTBI group. As with trait fatigue, control TC was predicted primarily by FP connectivity and one frontal edge of the CO (Figure 5). Interestingly, the CO edge (connecting right and left superior temporal nodes) most strongly predicting trait fatigue (β = −0.23) was also the only edge predicting state fatigue (β = −0.33) in the mTBI group, with weaker connectivity predicting less fatigue. This edge was not a significant predictor of either of the fatigue measures in the control group. The LSTG – RSTG connectivity decreased in the mTBI group from the beginning of the task to the 50% trial, increased considerably for the 75% first half, and then decreased again for the 75% second half. In the control group, the considerable increase in FC was at 50% first half (see Supplementary Figure 1). Thus, the timing of peak connectivity for this bilateral superior temporal edge differed considerably between groups. The delay in peak activity of this edge in the mTBI group may reflect an adaptive response cued by the need for increasing effort or the onset of fatigue as the task continued, resulting in increased CO connectivity to maintain task performance. Importantly, however, we note that there was suboptimal fit of the predictive models of TC with significant residual unexplained variance, particularly for the mTBI group. Further study of the brain network dynamics and their association with the behavioral task is warranted to improve the understanding of other aspects of brain organization (e.g., between-network interactions, whole-brain investigation of integration or segregation of community structure) that may better explain state fatigue. Also, addition of queries about the subjective judgment from each participant on the sense of effort or fatigue during task performance would be valuable.

The importance of bilateral superior temporal nodes for performance of the CE task may be unexpected, given that the temporal lobes are often thought to involve sensory processing of auditory stimuli. However, the superior temporal gyri, particularly on the right, have been found to be sensitive to prediction errors for ongoing performance in auditory (Kikuchi et al., 2019) and motor (Schmitter et al., 2021) tasks. It is known that when a participant is engaged in a task that requires monitoring of predictable sensory information, there is suppression of neural activity in the unimodal sensory cortex associated with task stimuli (e.g., auditory stimuli: auditory cortex; action: premotor areas). When there is perturbation in feedback during task performance (e.g., an unexpected tone is presented) suppression of the corresponding cortical region is released. A recent study has suggested that prediction errors are particularly linked to the right STG, as opposed to agency errors, or errors in implicit forward prediction, which is linked to the multimodal cortex (Kikuchi et al., 2019). This account was supported in an imaging study by Kikuchi et al. (2019) using auditory and visual stimuli. However, there are also data suggesting that the right STG may be involved in prediction errors regardless of stimulus type. For example, when a participant is performing a motor task for which continuous feedback is provided so that results of the movement are known in the moment, there is strong suppression of the right superior temporal gyrus (peak activity in Heschl’s gyrus, Schmitter et al., 2021). Thus, the complexity of the role of the superior temporal cortex in detecting prediction errors is elucidated for its role in multimodal processing. To explain the role of the STG in motor performance, Schmitter et al. (2021) highlight findings of the right STG in establishing representations of the limbs in space and matching that information to incoming visual feedback (Findlater et al., 2016; Limanowski and Friston, 2020) to support their findings. Thus, the role of at least the right STG in the CE task may be for the detection of prediction errors in the continuous maintenance of performance, even when the feedback on performance is in the sensorimotor domain.

The elevation and suppression of STG activity in task performance do not speak to its direct connectivity with other regions as investigated here. Connectivity between bilateral STG has been found to be sensitive to perturbations in task participation, again during a task dependent on motor and auditory inputs while producing voice (Flagmeier et al., 2014; Floegel et al., 2020). More specifically, Flagmeier et al. (2014) performed structural equation modeling to assess causal inferences about connectivity and found that the left STG modulates the activity of the right STG, possibly suppressing it during voicing. However, when the auditory input is perturbed so that the speaker receives aberrant feedback on their performance, connectivity changes such that the right STG inhibits the activity of the left. That shift in connectivity is associated with correction in voicing, updated based on the altered feedback on performance. Floegel et al. (2020) added and replicated the importance of frontal activity involved to change prediction and correct vocal production.

Together, the reports of these investigators suggest a potential role of the STG in performance of the CE task, and thus point to a hypothesis about the group differences observed between the mTBI and control groups. That is, the right STG–left STG edge demonstrated peak connectivity strength earlier, at lower effort levels, in the controls while it peaked later, at higher effort levels, in the mTBI group. While there was not an explicit perturbation in feedback for performance, the need to respond to changes in performance provided by internal feedback mechanisms during the CE task (e.g., recognition that maintaining effort is difficult, onset of fatigue) may serve as a perturbation. That is, when the task becomes more difficult, the participant must respond to the increasing sense of effort (perturbation) by updating their motor commands to attain or maintain pressure on the bulb. For the controls, this perturbation is evident as soon as the task becomes more challenging (as evidenced by their declining TC at 50%, Figure 1), but for the mTBI group, it is either that the need to increase effort level per task instruction is not perceived as a perturbation, or that the moment-to-moment sensorimotor feedback that they are receiving does not match their perceived sense of effort; thus, they do not change their performance (no change in TC, Figure 1). In either case, this is a prediction error, which Kikuchi et al. (2019) report as attributed to the STG. Thus, since this edge was predictive of both trait and state fatigue in the mTBI group, it may indicate that prediction errors in these participants, or rather the inability to detect prediction errors given that the beta value for this predictor is negative, are related to their higher report of fatigue on the FSS or their evidence of fatigue during the task. Further study is needed to determine whether errors in matching effort levels based on explicit task instruction, or errors in responding to internal feedback on a moment-to-moment basis, can be considered prediction errors.

In summary, the results reported here relate to the proposed roles of each network such that: (a) CO connectivity, associated with sustaining performance, was more relevant to performance and fatigue in the mTBI than the control participants (b), FP connectivity, associated with monitoring and flexibly changing according to cognitive goals, is weaker in mTBI relative to controls, and associated with trait and state fatigue only in the control group; and (c) the fluctuation of FP activity with TOT observed in the controls is very limited in the mTBI group, consistent with a mismatch between the effort demanded by the task and the effort expended. Given that the mTBI group demonstrated elevated CO connectivity across effort levels and TOT, did not modulate CO connectivity to attain task goals, and shifted connectivity to accommodate increasing effort level demands only late in the task, we hypothesize that the CO network is expending excess neural energy at the cost of detecting prediction errors in performance and updating forward predictions to complete the task. The control group, in contrast, demonstrated more involvement of the FP network for task modulation and successfully adjusted to task demands. We also hypothesize, based on these findings, that the fatigue in the mTBI group is related to adaptations to brain organization that are more reliant on the CO network to sustain effort than on the FP network, resulting in inefficient or ineffective detection of prediction errors.

## Data Availability Statement

The raw data supporting the conclusions of this article will be made available by the authors, without undue reservation.

## Ethics Statement

The one hundred and three individuals referred to the TBI service at the San Antonio Military Medical Center in San Antonio, Texas (SAMMC) provided written informed consent for participation per an approved and monitored Institutional Review Board (No. 3743378) and Human Research Protection Office at the United States Army Medical Department Medical Research and Materiel Command (No. A-17660).

## Author Contributions

AR, JL, and DR developed the methodology and designed the experiment and analysis. DT recruited and collected the fatigue measures and imaging data at SAMMC. All authors contributed to writing and editing the manuscript.

## Conflict of Interest

The authors declare that the research was conducted in the absence of any commercial or financial relationships that could be construed as a potential conflict of interest.

## Publisher’s Note

All claims expressed in this article are solely those of the authors and do not necessarily represent those of their affiliated organizations, or those of the publisher, the editors and the reviewers. Any product that may be evaluated in this article, or claim that may be made by its manufacturer, is not guaranteed or endorsed by the publisher.

## Footnotes

1
www.fil.ion.ucl.ac.uk/spm

2
www.nitrc.org
