# Supplementary material for: Cingulo-Opercular and Frontoparietal Network Control of Effort and Fatigue in Mild Traumatic Brain Injury
Source: Front Hum Neurosci. 2022 Feb 10;15:788091. doi: 10.3389/fnhum.2021.788091 (PMC8866657; doi:10.3389/fnhum.2021.788091)
Supplement: Supplementary file 1 [file Data_Sheet_1.docx]

| Network | ROI | MNI Coordinates | | | L/R | Region | Label |
| --- | --- | --- | --- | --- | --- | --- | --- |
|  |  | **X** | **Y** | **Z** |  |  |  |
| CO | 1 | -3 | 2 | 53 | L | Medial Frontal Gyrus | LmedFG |
| CO | 2 | 54 | -28 | 34 | R | Inferior Parietal Lobule | RIPL |
| CO | 3 | 19 | -8 | 64 | R | Middle Frontal Gyrus | RMFG |
| CO | 4 | -16 | -5 | 71 | L | Superior Frontal Gyrus | LSFG |
| CO | 5 | -10 | -2 | 42 | L | Cingulate Cortex | LCing |
| CO | 6 | 37 | 1 | -4 | R | Insula | RINS |
| CO | 7 | 13 | -1 | 70 | R | Superior Frontal Gyrus | RSFG |
| CO | 8 | 7 | 8 | 51 | R | Medial Frontal Gyrus | RmedFG |
| CO | 9 | -45 | 0 | 9 | L | Precentral Gyrus | LPcnt |
| CO | 10 | 49 | 8 | -1 | R | Superior Temporal Gyrus | RSTG |
| CO | 11 | -34 | 3 | 4 | L | Claustrum | LClaus |
| CO | 12 | -51 | 8 | -2 | L | Superior Temporal Gyrus | LSTG |
| CO | 13 | -5 | 18 | 34 | L | Anterior Cingulate Cortex | LACC |
| CO | 14 | 36 | 10 | 1 | R | Anterior Insula | RaINS |
|  |  |  |  |  |  |  |  |
| FP | 1 | -44 | 2 | 46 | L | Precentral Gyrus | LPcnt |
| FP | 2 | 48 | 25 | 27 | R | Inferior Frontal Gyrus (pars Triangularis) | RtriIFG |
| FP | 3 | -47 | 11 | 23 | L | Inferior Frontal Gyrus (pars Triangularis) | LtriIFG |
| FP | 4 | -53 | -49 | 43 | L | Inferior Parietal Lobule | LIPL |
| FP | 5 | -23 | 11 | 64 | L | Middle Frontal Gyrus | LMFG |
| FP | 6 | 58 | -53 | -14 | R | Inferior Temporal Gyrus | RITG |
| FP | 7 | 24 | 45 | -15 | R | Superior Frontal Gyrus (pars Orbitalis) | RorbSFG |
| FP | 8 | 34 | 54 | -13 | R | Superior Frontal Gyrus (pars Orbitalis) | RorbMFG |
| FP | 9 | 47 | 10 | 33 | R | Precentral Gyrus | RPcnt |
| FP | 10 | -41 | 6 | 33 | L | Precentral Gyrus | LPcnt2 |
| FP | 11 | -42 | 38 | 21 | L | Middle Frontal Gyrus | LMFG2 |
| FP | 12 | 38 | 43 | 15 | R | Middle Frontal Gyrus | RMFG |
| FP | 13 | 49 | -42 | 45 | R | Inferior Parietal Lobule | RIPL |
| FP | 14 | -28 | -58 | 48 | L | Inferior Parietal Lobule | LIPL2 |
| FP | 15 | 44 | -53 | 47 | R | Inferior Parietal Lobule | RIPL2 |
| FP | 16 | 32 | 14 | 56 | R | Middle Frontal Gyrus | RMFG2 |
| FP | 17 | 37 | -65 | 40 | R | Angular Gyrus | RAG |
| FP | 18 | -42 | -55 | 45 | L | Inferior Parietal Lobule | LIPL3 |
| FP | 19 | 40 | 18 | 40 | R | Middle Frontal Gyrus | RMFG3 |
| FP | 20 | -34 | 55 | 4 | L | Middle Frontal Gyrus | LMFG2 |
| FP | 21 | -42 | 45 | -2 | L | Middle Frontal Gyrus (pars Orbitalis) | LorbMFG |
| FP | 22 | 33 | -53 | 44 | R | Angular Gyrus | RAG2 |
| FP | 23 | 43 | 49 | -2 | R | Middle Frontal Gyrus (pars Orbitalis) | RorbMFG2 |
| FP | 24 | -42 | 25 | 30 | L | Inferior Frontal Gyrus (pars Triangularis) | LtriIFG2 |
| FP | 25 | -3 | 26 | 44 | L | Superior Medial Frontal Gyrus | LsupmedFG |

Supplemental Table 1. Nodes making up the cingulo-opercular (CO) and frontoparietal (FP) networks based on the original regions of interest (ROI, numbered as in Figure 6) from Power et al. (2011). Regions and labels were determined using the Talairach Daemon (Lancaster et al., 2000). L = left hemisphere, R = right hemisphere.


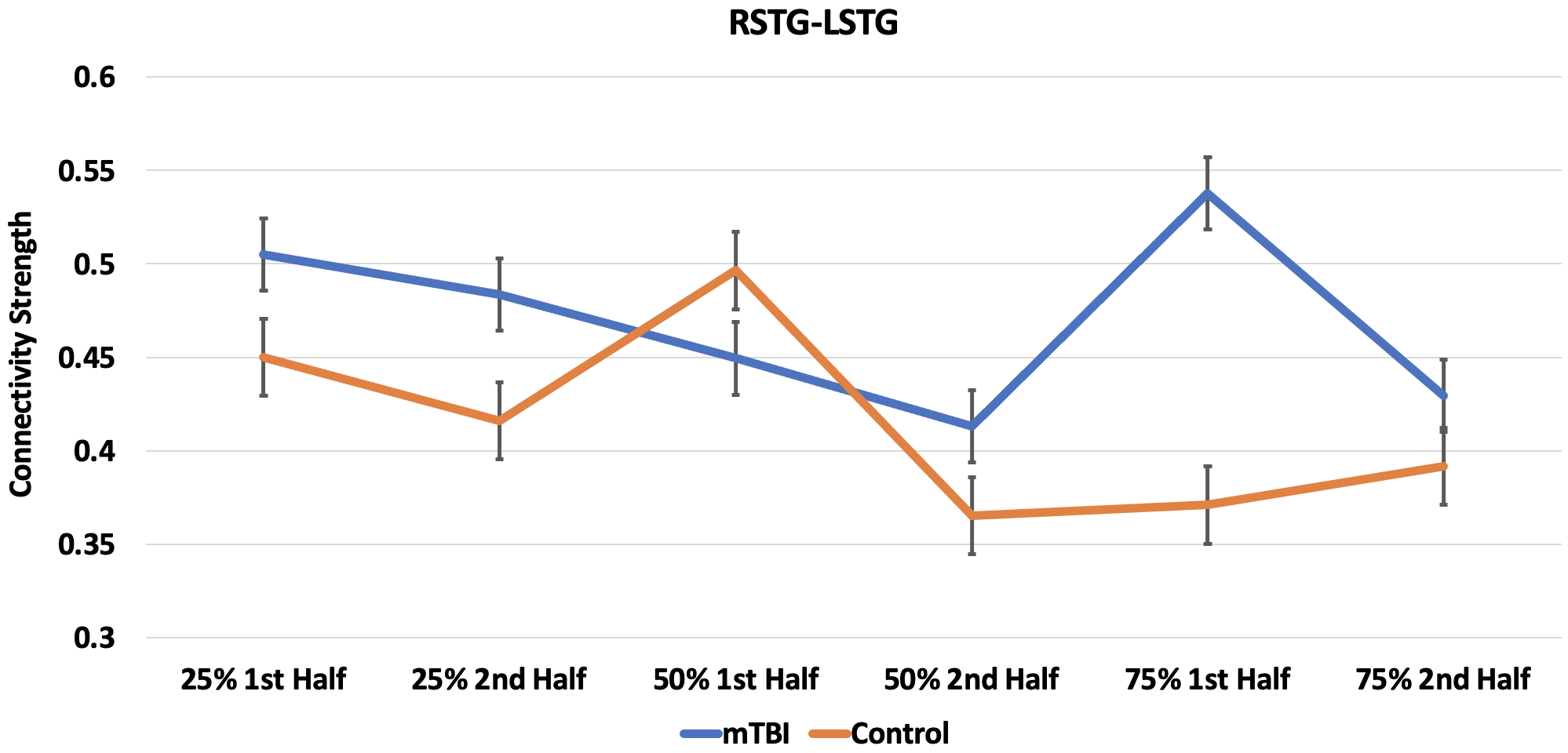


**Supplemental Figure 1.** Functional connectivity of the right superior temporal gyrus (RSTG) – left superior temporal gyrus (LSTG) edges of the CO reduced in both groups from the beginning of the task but peaked in the Control group at 50% and in the mTBI group at 75%. The peaking of connectivity may be an indication of a prediction error, or a need to increase effort based on feedback about performance, which was evident earlier in the task, when it was conceivably becoming more difficult at 50% in the Controls. In contrast, this prediction error did not occur until the 75% effort level condition in the mTBI group. Error bars = standard error.
